# Self‐Assembled Graphene‐Based Architectures and Their Applications

**DOI:** 10.1002/advs.201700626

**Published:** 2017-11-30

**Authors:** Zhongke Yuan, Xiaofen Xiao, Jing Li, Zhe Zhao, Dingshan Yu, Quan Li

**Affiliations:** ^1^ Key Laboratory for Polymeric Composite and Functional Materials of Ministry of Education Key Laboratory of High Performance Polymer‐based Composites of Guangdong Province School of Chemistry Sun Yat‐sen University Guangzhou 510275 China; ^2^ Liquid Crystal Institute and Chemical Physics Interdisciplinary Program Kent State University Kent OH 44242 USA

**Keywords:** catalysis, energy conversion, energy storage, hybrid structures, optoelectronics, self‐assembled graphene

## Abstract

Due to unique planar structures and remarkable thermal, electronic, and mechanical properties, chemically modified graphenes (CMGs) such as graphene oxides, reduced graphene oxides, and the related derivatives are recognized as the attractive building blocks for “bottom‐up” nanotechnology, while self‐assembly of CMGs has emerged as one of the most promising approaches to construct advanced functional materials/systems based on graphene. By virtue of a variety of noncovalent forces like hydrogen bonding, van der Waals interaction, metal‐to‐ligand bonds, electrostatic attraction, hydrophobic–hydrophilic interactions, and π–π interactions, the CMGs bearing various functional groups are highly desirable for the assemblies with themselves and a variety of organic and/or inorganic species which can yield various hierarchical nanostructures and macroscopic composites endowed with unique structures, properties, and functions for widespread technological applications such as electronics, optoelectronics, electrocatalysis/photocatalysis, environment, and energy storage and conversion. In this review, significant recent advances concerning the self‐assembly of CMGs are summarized, and the broad applications of self‐assembled graphene‐based materials as well as some future opportunities and challenges in this vibrant area are elucidated.

## 1 Introduction

Graphene, a 2D planar honeycomb array formed by monolayer carbon atoms, has gained a surge of interest due to its unique 2D structure and remarkable electronic, thermal, and mechanical properties desirable for various potential technical applications.^1^, ^2^, ^3^, ^4^, ^5^, ^6^, ^7^ To date, graphene has been fabricated by using various methods including mechanical^1^ or chemical exfoliation,^5^ chemical vapor deposition (CVD),^7^ and epitaxial growth.^8^ Pristine defect‐free graphene cannot be well dispersed in water or in a range of various organic solvent.^8^ Because of graphene's poor processability, it is difficult to employ traditional processing methods to build these 2D building blocks into desirable structures and eventually into a functional system that is of great importance to exploit useful properties of the individual graphene nanosheets (NS) for practical macroscopic applications. In sharp contrast, graphene oxides (GOs), reduced graphene oxides (rGOs), and the related derivatives, generally defined as chemically modified graphenes (CMGs), are superior alternatives because of the following advantages: (i) the preparation for CMGs is usually performed by a combined chemical oxidation and exfoliation process using pristine graphite as starting precursors without any particular instruments such as the CVD system or the tedious mechanical exfoliation process, and therefore is low cost and easy for the mass production; (ii) some CMGs such as GOs and chemically modified rGOs bear rich functional groups including hydroxyl, carboxyl, epoxy, and carbonyl groups, which could reduce the intersheet stacking and thereby render their good dispersion in solutions;^9^ (iii) CMGs usually act like both molecules and colloid surfactants, making them unique 2D building blocks favorable for the assembly with themselves and other inorganic/organic components by noncovalent forces;^9^ (iv) the multiple functional groups and conjugated carbon basal plane of CMGs afford a lot of opportunities for chemical functionalization to endow them with improved properties and new functions. Therefore, CMGs hold great potential in a series of various applications including those in electronics, optoelectronics, electrocatalysis/photocatalysis, energy conversion and storage systems, environment, and so on, and have attracted significant interest especially in the fields of materials and chemistry.^8^, ^9^, ^10^

On the other hand, it has been recognized that translating individual graphene sheets into well‐defined hierarchical architectures and ultimately into high‐performance functional systems will pave the way to meet the practical demand of various applications that require bulk graphene materials. Self‐assembly has been considered as one of the most promising approaches to construct advanced graphene‐based functional materials, which involves the organization of different building blocks into complex superstructures of various scales through noncovalent interactions. By virtue of a range of noncovalent forces such as hydrogen bonding, van der Waals interactions, metal‐to‐ligand bonds, electrostatic attraction, hydrophobic–hydrophilic interactions, and π–π interactions, CMGs with multiple functional groups are desirable for the assembly with themselves and a wide variety of organic and/or inorganic functional components which can yield hierarchical nanostructures and macroscopic composites endowed with unique structures, properties and functions for broad practical applications.^9^ Accordingly a variety of graphene‐based hierarchical nanostructures and functional hybrid architectures have been produced via the self‐assembly of CMG building blocks.

In this review, we provided a broad overview covering the latest assembly strategies that have been explored to build a variety of graphene‐based nanostructures as well as macroscopic architectures including macroscopic fibers, thin films, membranes, papers, and porous networks. Furthermore, we also highlight some typical examples of the potential technical applications of self‐assembled graphene‐based materials. Lastly, we elucidate some potential opportunities and challenges in this ever growing field. We anticipate that this review and ongoing efforts in this emerging field will provide useful guidelines to scientists toward highly efficient production of self‐assembled CMG‐based structures and their diverse real‐world applications.

## 2 State‐of‐the‐Art Self‐Assembly Strategies of Graphene Architectures

The self‐assembly for CMGs usually occurs in a solution or at an interface with π–π stacking, hydrophobic/hydrophilic interactions between each of their basal planes, or electrostatic interactions of the functional groups as the main driving forces. Because of their attractive 2D structural characteristics including high aspect ratio and large specific surface area as well as their unique supermolecular properties, CMGs are favorable for the assembly into different graphene architectures, such as large‐area thin films, paper‐like membranes, 3D porous network structures, 1D macroscopic fibers. To date, several effective approaches have been established to assemble these 2D building blocks either in solution or at the interface, including layer‐by‐layer (LbL) assembly technique, Langmuir–Blodgett (LB) deposition, flow‐directed, evaporation‐induced, or interface‐induced self‐assembly, and template‐directed/space‐confined assembly. This section will present the state‐of‐the‐art of the strategies for the self‐assembly of graphene nanostructures and architectures.

### 2.1 Langmuir–Blodgett Method

LB assembly is well known as a robust and powerful approach for translating 2D building blocks into high‐quality thin films with ordered microstructures and desired functionality at the molecular scale by promotion of lateral packing and well‐controlled compression at the air/liquid interface.^11^ It was suggested that the marginal electrostatic repulsion between single graphene layers inhibits their overlap when compressed at the air–liquid interface, thereby yielding an ordered monolayer film on hydrophilic substrate. CMGs are featured as planar macromolecules with large aspect ratios. To exploit practical applications of graphene in nanoscale electronics, the LB technique is an ideal approach to achieve flat, large‐area, single‐ or multilayered graphene films, since it is capable of achieving fine control of the monolayer thickness and homogeneous deposition as well as the formation of multilayers with various layer compositions.

**Figure 1:**
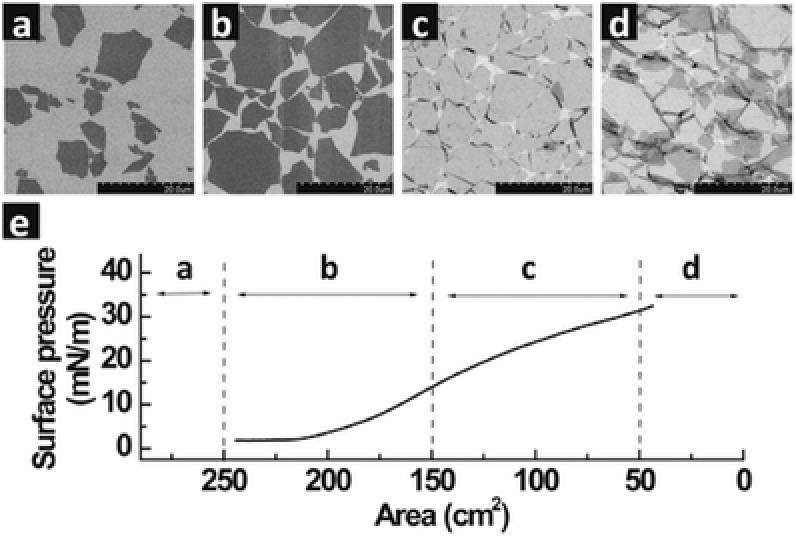
Langmuir–Blodgett assembly of GO single layers. a–d) SEM images showing the collected GO monolayers on a silicon wafer at different regions of the isotherm. The packing density was continuously tuned: a) dilute monolayer of isolated flat sheets, b) monolayer of close‐packed GO, c) overpacked monolayer with sheets folded at interconnecting edges, and d) over packed monolayer with folded and partially overlapped sheets interlocking with each other. e) Isothermal surface pressure/area plot showing the corresponding regions (panels (a)–(d)) at which the monolayers were collected. Scale bars in panels (a)–(d) represent 20 µm. Reproduced with permission.^14^ Copyright 2009, American Chemical Society.

Fine dispersion of the building blocks in solutions is a key prerequisite for LB assembly. Considering that amphiphilic GO sheets are able to be readily dispersed in aqueous solution and certain organic solvents, GO has now been considered as a popular precursor for fabricating large‐area graphene films.^12^, ^13^ Huang and co‐workers demonstrated that stable monolayer GO films could be achieved without using any stabilizing agent by the LB technique, as shown in **Figure** **1**.^14^ The monolayers are easy to form a large‐area, and flat GO film with adjustable density from dilute close‐packed to overpacked monolayers. An ordered monolayer film constructed by GO decorated with octadecyl ester of rhodamine B was assembled onto a hydrophilic substrate by using the LB technique as well.^15^ By varying the pH and the GO concentration in the water subphase, the thin‐film architecture can be readily tuned. In addition to hydrophilic GO sheets, the LB technique was also employed to assemble hydrophobic rGO sheets produced by various methods into large‐area conducting film with fine control over the thickness on different substrates.^16^, ^17^, ^18^

Traditionally, the LB method has often been used for the deposition of 2D structures. More recently, by exploring the conventional dipping mechanism in an LB trough, Vengadesh and co‐workers have built an unconventional LB assembly method, which compresses the monolayer beyond the collapse pressure state to produce the 3D porous graphene films.^19^ The high porosity and the tunable roughness of rGO films from nanoscale to macroscale can be well engineered during the deposition process.

Patterning of graphene films on different substrates has tremendous impact in a range of fields like nanoelectronics. Kumar and co‐workers successfully utilized the LB deposition method in conjunction with selective N_2_‐plasma‐engaged treatment of the SiO_2_/Si substrates to transform GO sheets with nonuniform size into a large‐area and highly ordered array.^20^ This method enables the formation of various patterned graphene thin films with controllable dimensions.

### 2.2 LbL Assembly Method

The LbL method, which involves electrostatic interactions and hydrogen bonding as attractive forces for sequential adsorption of oppositely charged components, is an appealing approach for the fabrication of highly tunable and multilayered graphene architectures, as it enables the nanoscale‐level controlled thickness and composition, as well as the tunable physicochemical properties of the multilayered films, by adjusting some parameters including the ionic strength, pH values, and concentration.^21^

**Figure 2:**
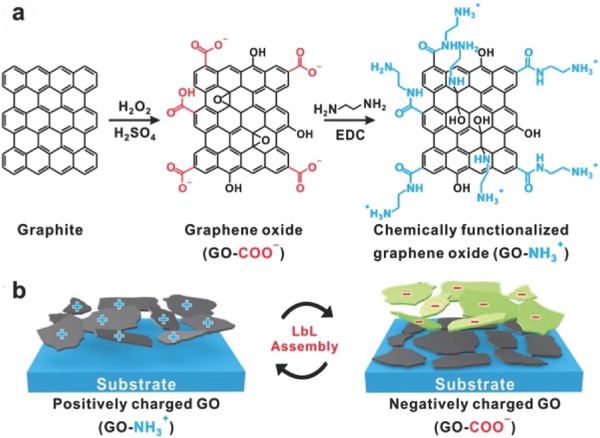
Schematic illustration of a) synthesis of oppositely charged graphene and b) the generation of graphene thin films using an electrostatic layer‐by‐layer (LbL) assembly between oppositely charged graphene nanosheets and subsequent thermal treatment. Reproduced with permission.^26^ Copyright 2011, American Chemical Society.

Up to now, the assembly of a series of different CMGs into the multilayered architectures by LbL assembly has been demonstrated, yielding highly controllable and conformal thin films on different substrates.^21^, ^22^, ^23^, ^24^, ^25^ To achieve an effective LbL multilayered assembly, a critical requirement is that two oppositely charged components should be well dispersed in solution. Yoo and co‐workers prepared well‐dispersed aqueous solutions of negatively charged graphene nanosheets (rGO–COO^−^) by chemical reduction of GO and positively charged graphene nanosheets (rGO–NH_3_^+^) by the modified thionyl chloride chemistry. Therefore, through alternating LbL assembly between rGO–COO^−^ and rGO–NH_3_^+^, homogeneous multilayered all‐graphene thin films were created based on electrostatic interaction (**Figure** **2**). Precise control of the thin‐film thickness was achieved by tuning the stacking numbers. The LbL‐assembled rGO films showed easily modulated light transmittance and sheet resistance by varying the bilayer number, which is highly dependent on the multilayer thickness, via nanoscale engineering of the assembly. Further thermal treatment leads to enhanced electrical conductivity. Considering that all processing steps are carried out in aqueous environments, the LbL method is environmentally friendly and compatible with industrial scale‐up processes.^26^

Zhu and Tour also produced dispersions of the positively charged amino‐functionalized graphene nanoribbons (fGNRs) in N,N‐dimethylformamide (DMF)/HCl and the negatively charged fGNRs with sulfonic group in water. Even though the LbL deposition was alternatively performed in water and DMF/HCl, uniform GNR films can also be produced on quartz and silicon wafer substrates with controllable thicknesses. When these GNR films were used for bottom‐gated field‐effect transistors (FETs), the device exhibited mobilities of 0.1–0.5 cm^2^ V^−1^ s^−1^.^27^

Grunlan and co‐workers reported the fabrication of a multilayered thin film, in which polyvinylpyrrolidone‐stabilized graphene (PVP‐G) platelets and poly(acrylic acid) (PAA) are alternately deposited using hydrogen‐bonding‐assisted LbL assembly. Such a multilayered thin film showed an increase of the elastic modulus (*E*) of a polymer multilayered thin film by 322% (from 1.41 to 4.81 GPa), while maintaining a visible light transmittance of ≈90%.^82^

Furthermore, LbL assembly enables the preparation of not only conformal 2D thin films on a 2D surface but also 3D objects. By repeatedly assembling the components of rGO–NH_3_^+^ and rGO–COO^−^ onto sacrificial polystyrene (PS) colloidal particles, hollow capsules of CMGs were constructed based on the electrostatic attraction by the LbL technique. Expanding this approach, a new functionality such as gold nanoparticles (NPs) can be introduced into a hollow graphene capsule. The approach may open up new avenues for constructing hollow graphene structures with multiple functionalities.^24^

### 2.3 Flow‐, Evaporation‐, and Interface‐Induced Self‐Assembly

**Figure 3:**
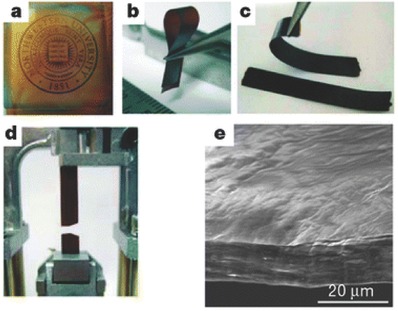
Morphology and structure of GO paper. a–d) Digital camera images of GO paper: a) 1 mm thick; b) folded, 5 mm thick semitransparent film; c) folded, 25 mm thick strip; d) strip after fracture from tensile loading. e) Side‐view SEM images of the 10 mm thick sample. Reproduced with permission.^28^ Copyright 2007, Nature Publishing Group.

Flow‐directed self‐assembly, also called shearing field‐assisted alignment of suspensions, has been widely employed as a cheap yet efficient method to produce various foil‐like and inorganic paper‐like films based on exfoliated vermiculite or mica platelets. Pioneering work to extend this method for the assembly of GO was performed by Ruoff and co‐workers^28^ In this respect, the aqueous GO solution consisting of individual nanosheets was first prepared by chemical exfoliation from graphite powder followed by the ultrasonication in water, and the resultant aqueous GO was filtrated through a porous membrane filter, finally yielded GO paper with the thickness of 1–30 µm. Since vacuum filtration induces a directional flow, individual GO sheets were subjected to a near‐parallel arrangement, affording a free‐standing membrane consisting of a well‐packed layered architecture after air‐drying (**Figure** **3**). The produced GO paper has good flexibility and excellent mechanical performance possessing a tensile strength up to ≈80 MPa with Young's modulus up to ≈32 GPa, which outperforms many other paper‐like or foil‐like materials. It is suggested that hydrogen bonding and strong van der Waals forces occurred within the GO nanosheets^28^ are responsible for the remarkable mechanical performance of the GO paper.

Though the GO papers often possessed great mechanical strength, they still suffered from low electric conductivity and poor thermal stability, constraining their broad applications. Following on the Ruoff's work, Li et al. prepared rGO papers by using similar vacuum filtration of readily dispersed aqueous rGO stabilized by electrostatic repulsion. The obtained layered rGO paper showed a shiny metallic luster while possessing a tensile strength of 35 GPa similar to that obtained for a GO paper. Moreover, the rGO paper exhibited significantly enhanced electrical conductivity of up to 7200 S m^−1^ and thermal stability. By modulating the volume and concentration of the rGO dispersion, fine control over the thickness of the rGO paper could be achieved with flow‐induced assembly technique.^29^ This strategy were also applied to the preparation of transparent graphene films with nanoscale thicknesses.^30^, ^31^, ^32^, ^33^ For instance, Eda et al. fabricated large‐area GO films with controllable thickness, varying from a monolayer to multiple layers through the vacuum filtration strategy using a diluted GO aqueous dispersion.^33^ Combined chemical reduction and thermal treatment can transform the insulated GO thin films into highly conductive rGO films desirable for use in transparent conductive electrodes and flexible electronics such as transistors.

In fact, flow‐induced assembly method was also extended to other readily dispersed graphene sheets either in aqueous solution or in some organic solvents such as propylene carbonate.^34^, ^35^, ^36^, ^37^, ^38^, ^39^, ^40^ For example, through vacuum filtration, 1‐pyrenebutyrate‐modified rGO aqueous dispersion could form CMG films with high conductivity and good mechanical performance.^34^ rGO sheets produced by thermal reduction can be dispersed in propylene carbonate, which was utilized for assembly into paper‐like membranes with good conductivity and thermal stability.^40^ Further investigation on the assembly mechanism for the paper‐like GO thin films indicated the occurrence of graphene gelation at the solution/filter membrane interface during the course of vacuum filtration.

Self‐assembly at the phase interface is also regarded as a promising approach for the construction of paper‐like graphene films. In this regard, GO with an amphiphilic character is able to generate a packing in an oriented fashion at the air–liquid interface. Sequential heating led to the formation of a condensed film of GO at the air–liquid interface. After subjecting to drying and further reduction, a semitransparent and self‐supporting membrane with high mechanical flexibility and a tunable thickness of 0.5–20 µm was obtained.^41^ Within the presence of a handful of ethanol, adjustable transmittance and resistivity of the GO films were readily realized at the water/pentane interface during the course of the pentane evaporation.^42^

Evaporation has been employed to prepare GO paper, since it is capable of triggering the directional flow of the solvent.^41^ Compared with the conventional vacuum filtration method, the evaporation‐triggered self‐assembly method can save time. A GO paper with the thickness as thin as several micrometers was achieved by self‐assembly induced by the evaporation, which took only 10–40 min at 80 °C, while flow‐directed self‐assembly requires 12–24 h for obtaining a GO paper with the identical thickness, yet it led to superior mechanical performance relative to the evaporation‐induced assembly. In conjunction with in situ chemical reduction, evaporation‐triggered assembly can be applicable to fabricate transparent conductive graphene‐based films.^43^ By controlling the evaporation for the GO suspension in a special vacuum concentrator, either 3D GO sponges or papers can be selectively produced. An rGO sponge can be obtained by subsequent thermal treatment at high temperature.^44^

### 2.4 Template‐Directed Self‐Assembly and Hydrothermal Processes

Self‐assembly of graphene into monolithic macroscopic superstructures with 3D networks can largely translate the admirable characteristics of individual graphene into resultant macrostructures, thus enabling applications that require high‐conductivity, large‐surface‐area, and self‐standing structures. To date, 3D self‐assemblies for CMG‐based architectures in the form of hydrogels, aerogels, and foams have been realized by template‐directed assembly and hydrothermal processes.^45^, ^46^

**Figure 4:**
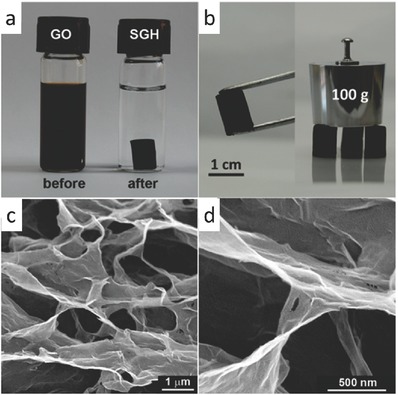
a) Photographs of a 2 mg mL^−1^ homogeneous GO aqueous dispersion before and after hydrothermal reduction. b) Photographs of a strong graphene hydrogel allowing easy handling and weight support. c,d)Scanning electron microscope (SEM) images with different magnifications of the graphene hydrogel's interior microstructures. Reproduced with permission.^46^ Copyright 2010, American Chemical Society.

Pioneering work in this area was conducted by Shi and co‐workers, who effected the effective self‐assembly of graphene hydrogels via a hydrothermal processing. In this regard, by a convenient one‐step hydrothermal process of a highly concentrated GO aqueous dispersion using a sealed Teflon‐lined autoclave at 180 °C for 12 h, 3D porous graphene hydrogel was generated, which consisted of a highly interconnected 3D graphene network (≈2 wt%) filled with water (≈98 wt%) with various pore sizes from nanometers to several micrometers, and pore walls constructed by the graphene stacking.^46^ As proposed by Shi and co‐workers, when GO was subjected to hydrothermal reduction, the oxygenated functional groups on graphene sheets significantly decreased, and the π‐conjugation was largely restored. When the GO concentration was sufficiently high, the π–π stacking interaction in conjunction with decreased hydrophilicity and electrostatic repulsion triggered flexible rGO sheets to partially overlap and interlock with each other to generate enough cross‐linking sites for building a 3D porous framework with entrapped water by the residual oxygen‐carrying groups. By contrast, when the GO concentration was too low, the cross‐linking became less likely to occur in time owing to relative few opportunities for the contact between the dispersed graphene sheets in the aqueous solution. Therefore, GO sheets were converted into graphene aggregates, as shown in **Figure** **4**. Qiu and co‐workers developed an ultralow density and compressible aerogels of graphene by a hydrothermal‐induced assembly in conjunction with functionalization–lyophilization–microwave treatment. The resulting aerogels have shown an ultralow density of only 3 mg cm^−3^, but the original structure can recover with no explicit fracture after 90% compression.^47^

Hollow CMG structures can be produced through template‐directed assembly. For instance, by utilizing a soft template based on the water–oil interface from a water/oil emulsion, Guo et al. assembled amphiphilic GO sheets into hollow GO spheres with diameters ranging from 2 to 10 µm.^48^ The oxidation degree of GO affected its self‐assembly behavior as well as the structures and sizes of the resulting microspheres. The vigorous oxidation can yield smaller and more hydrophilic GO nanosheets, thereby generating smaller graphene microspheres with hollow structures. In such a system, the flexible amphiphilic GO nanosheets can reaggregate at the water–oil interface in the absence of extra surfactant, while the compact shell of graphene is achieved mainly by interactions between individual graphene sheets such as van der Waals forces and/or electrostatic attraction and/or hydrogen bonding interaction. Diphenylalanine peptide nanowires were also employed as a solid template to incorporate GO sheets into core–shell hybrid architecture by virtue of electrostatic interaction. Thickness modulation for the shell of GO was achieved by adjusting the pH during self‐assembly processes and thereby tuning the interaction between GO and peptide templates. Further calcination treatment will remove the peptide core and generate hollow rGO structures with large surface area and excellent chemical/thermal stability.^49^

### 2.5 Spinning and Space‐Confinement Self‐Assembly

**Figure 5:**
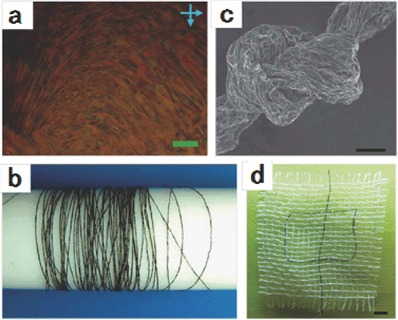
a) Polarized optical microscopy image of GO LCs with a volume fraction of 0.76%. b) A long GO fiber wound on a Teflon drum with a diameter of 2 cm. c) SEM image of a knotted GO fiber. d) A Chinese character pattern knitted in the cotton network (white) using two graphene fibers (black). Reproduced with permission.^50^ Copyright 2011, Nature Publishing Group.

In comparison with the 2D and 3D self‐assemblies of CMG sheets discussed in the previous section, integrating CMG sheets into 1D macroscopic fiber is a challenging task due to the irregular shape and nonuniform size together with strong stacking tendency of graphene layers. Two major barriers for making macroscopic graphene fibers were the poor dispersion of graphene and the lack of efficient assembly strategy.^50^, ^51^, ^52^ The 2D topology of graphene satisfies the basic asymmetrical structural factor for liquid crystals (LCs),^53^, ^54^, ^55^, ^56^, ^57^, ^58^, ^59^ and the findings of crystalline behavior of graphene and its derivatives offer a straightforward yet effective LC‐based wet‐spinning method for making macroscopic graphene fibers. Xu and Gao first reported continuous and neat graphene fibers via a wet‐spinning process built on unique LC character of graphene (**Figure** **5**). By injecting a condensed GO dispersion into the coagulation bath, the first meter‐long, polymer‐free GO fibers, as demonstrated by Xu and Gao showed great mechanical performance (102 MPa strength and 5.4 GPa modulus) and greater fracture elongations than most of the carbon nanotube (CNT) fibers. Importantly, after the reduction in hydroiodic acid, the rGO fibers presented a good conductivity of 2.5 × 10^4^ S m^−1^ and even greater mechanical performance (140 MPa strength and 7.7 GPa modulus) while maintaining the remarkable fracture elongation (5.8%). The strength enhancement of graphene fibers was attributed to the increased intersheet interactions of graphene, originating from the decreased interlayer distance within the reduced fibers (as shown in Figure 5).^50^

Taking advantage of the solution‐based wet‐spinning technique, the morphology of the cross section of the fibers could be easily controlled to fulfill different requirements for multifunctional applications. By modulating the coagulation process and nozzle types in the spinning process, porous and coaxial structures were introduced into the graphene fiber structures. Gao and co‐workers, employing liquid nitrogen as the coagulation bath with subsequent freeze‐drying, prepared fiber‐like graphene aerogel with a core–shell structure (porous core and compact shell). The coaxial layered architecture with an oriented packing of GO sheets yielded a large specific tensile strength of 188 kN m kg^−1^ together with a compression strength of 3.3 MPa, to a certain extent, compromising the strength and porosity.^52^

Space‐confined hydrothermal processing was found to be even a handier method compared to the wet‐spinning process. In short, the one‐step hydrothermal fabrication procedure was achieved by baking a hydrothermal reactor with space‐confinement fillers containing cylindrical channels with the aqueous GO dispersion. It was found that the GO sheets aligned along the fiber axis, which was ascribed to the shear force induced by capillary and the tensile force due to surface tension. For instance, Qu and co‐workers utilized a glass microtube as the space‐confined microreactor. The concentrated aqueous GO suspension was filled into the glass microtube, followed by subsequent thermal treatment in a two‐end‐sealed microtube at 230 °C for 120 min. A neat graphene fiber was then produced, showing a tensile strength of 180 MPa and a much higher strength up to 420 MPa achieved after further vacuum thermal treatment of the fiber at 800 °C. This annealing effect could further improve the strength and toughness of the CNT fiber by rendering extra covalent bonding at the interfaces.^51^

## 3 Self‐Assembled Graphene‐Based Hybrid Structures

As mentioned in the above section, self‐assembly has proved an effective method to construct a variety of pure graphene‐based architectures. Actually, CMGs can also be integrated with other inorganic/organic components to form functional hybrid architectures with desirable morphology, structure, and properties. CMG‐built hybrid structures are of great significance due to intriguing features which not only inherited intrinsic characteristics of individual component but also generated new properties and functions by the synergistic effect. This following section describes the built‐up of CMG‐based architectures including hybrid structures by using the above‐mentioned self‐assembly techniques.

### 3.1 Hybrid Structures with Carbon Nanomaterial

Carbon nanomaterials other than graphene, such as fullerenes and CNTs, possess many attractive features, and so does their assembled architecture with graphene in all three dimensions. Because of the strong interactions between assembled components (strong π–π interaction, van der Waals force, large interaction surfaces between sheets, corrugation at atomic scale, and winkled morphology at the sub‐micrometer scale) and the synergic effect of individual nanocarbon components, the hybridization of two or more constituents usually exhibits more advanced properties than a single component. Thus, such nanocarbon/graphene hybrids have attracted increasing interest during the past decades, especially for hybridization with assembled architectures from macroscopic 1D fibers to 3D structures.^60^, ^61^

**Figure 6:**
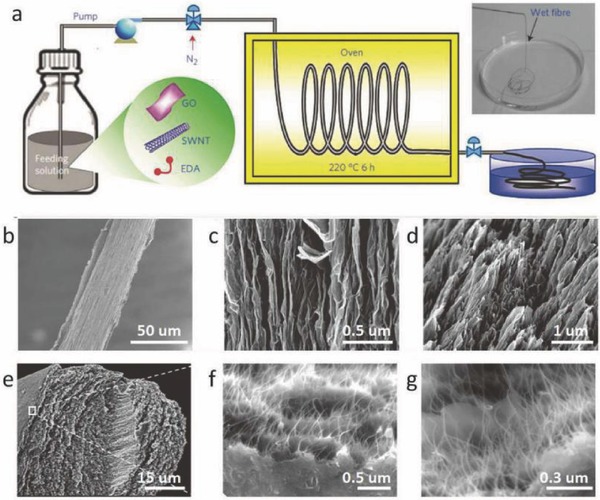
a) Schematic of the synthesis of carbon hybrid microfibers. The inset showed the optical image of the wet fiber. b–d) SEM images of the full view, outer surface and fracture end area of the rGO fiber. e–g) SEM images of the cross section of CNT–rGO fiber. The square area in panel (e) is highlighted in panel (f). Reproduced with permission.^63^ Copyright 2014, Nature Publishing Group.

1D hybrid nanocarbon fibers can be prepared from CNTs and graphene or their derivatives via a similar wet‐spinning process for enhanced mechanical and electrical performance. For instance, Kim and co‐workers synthesized continuous hybrid fibers by mixing rGO and single‐walled carbon nanotubes (SWCNTs) into a stable dispersion with the surfactant sodium dodecyl benzene sulfonate, followed by wet spinning into a poly(vinylalcohol) (PVA) coagulation bath. Such hybrid fibers showed admirable mechanical performance with gravimetric toughness as high as 1000 J g^−1^. Such outstanding toughness was attributed to a framework consisting of partially aligned CNTs and rGO sheets during solution spinning.^62^ In our earlier effort, we proposed a scalable and continuous hydrothermal method for the synthesis of GO/CNT hybrid fibers by integrating GO sheets and acid‐oxidized SWCNTs within the presence of ethylene diamine (EDA) to an uniform aqueous suspension with a subsequent in situ hydrothermal process (**Figure** **6**). The hierarchically structured microfiber consisting of an interconnected aligned SWCNTs' network with interposed N‐doped rGO sheets yielded an ultralarge specific capacitance up to 305 F cm^−3^ in a fiber‐shaped supercapacitor (SC).^63^ Foroughi et al. reported another CNT/graphene composite yarn with the electrical conductivity as high as 900 S cm^−1^ considerably higher than that of pristine CNT yarns.^64^ This value was 400% and 1250% larger than those achieved for graphene paper or pristine CNT yarns, respectively. The enhanced conductivity is credited to the increased density of states around the Fermi level by two orders of magnitude as well as the decreased hopping distance by an order of magnitude by introducing graphene into CNT yarns, which results in more delocalized charge carriers and significantly increased conductivity of the CNT/graphene yarns.^64^ Recently, Filleter and co‐workers reported an improvement in the mechanical performance of commercially available CNT yarns/fibers by the introduction of interlocking GO sheets by immersing CNT yarns into a GO solution with subsequent air‐drying. It was found that GO could serve as an effective bridge that interlocks neighboring CNTs, as illustrated in Figure 6, and enhance load transfer within the whole yarn with further improvement in the macroscopic mechanical performance.^65^

2D macroscopic graphene hybrid films with excellent mechanical strength, superb electric conductivity, and excellent chemical stability have been widely studied for transparent conductive films,^66^, ^67^, ^68^, ^69^ flexible SC electrodes,^70^ sensors,^71^ and other applications.^72^ For instance, Gao and co‐workers designed graphene/multiwalled carbon nanotube (MWCNT) membranes by assembling refluxed GO and MWCNTs on a porous substrate via simple filtration‐assisted assembly^73^ with an increased water flux compared to the previously reported neat graphene nanofiltration membrane.^74^ In this composite film, MWCNTs were applied as “nanoedge” to expand the interlayer spacing between neighboring graphene sheets. LbL electrostatic self‐assembly as a versatile fabrication method can also be utilized for graphene‐based composite films. Our group also reported the self‐assembly of positively charged water‐soluble poly(ethylene imine) (PEI)‐modified graphene sheets with negatively charged acid‐oxidized MWCNTs. The resulted composite films were found to have an interconnected carbon network structures with desirable porosity, which holds promise for SC electrodes.^75^

Besides CNTs, other nanocarbon materials can also form hybrid films with graphene with the filtration method. Zhang and co‐workers reported mesoporous carbon nanosphere/graphene hybrid films by the filtration of a mixture of a dispersion of mesoporous carbon nanosphere with a 200 nm porous polytetrafluoroethylene (PTFE) membrane to produce a graphene/mesoporous carbon sphere film, showing higher specific capacitance per surface area (0.36 F m^−2^) than that of a powdery graphene/mesoporous carbon sphere composite (0.23 F m^−2^).^76^

**Figure 7:**
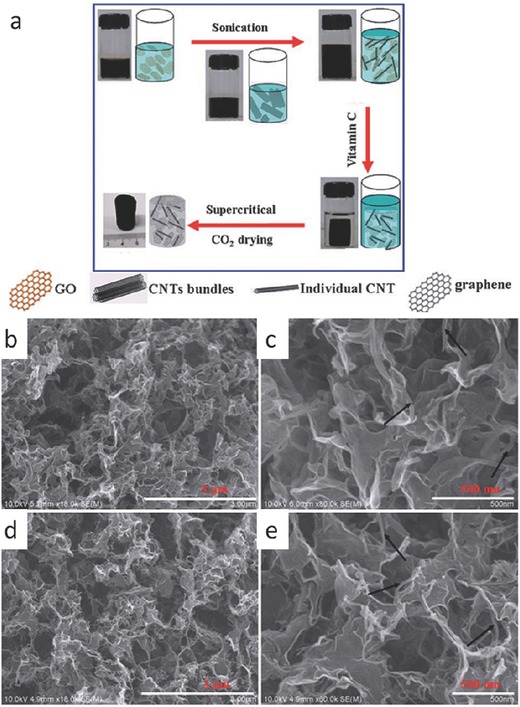
a) Schematic diagram for green synthesis of the graphene–CNT hybrid aerogels. SEM images of b,c) the resulting graphene–MWCNT and d,e) graphene–c‐MWCNT hybrid aerogels. Reproduced with permission.^77^ Copyright 2012, Royal Society of Chemistry.

Self‐assembly of graphene hybrids into monolithic macroscopic superstructures with 3D networks can largely translate the admirable features of the individual component into the resultant macrostructures and enriching applications that demand high conductivity, large surface area, and self‐standing structures. It has therefore attracted significant interest in the past years. To date, some examples regarding the fabrication of graphene/carbon composite‐based 3D architectures have been demonstrated. For instance, Zhang and co‐workers reported a green process for making graphene/MWCNT hybrid aerogels in a pioneering work. This process was established with the homogeneous mixing of predispersed GO sheets and MWCNTs, chemical reduction‐induced hydrogel formation, and subsequent supercritical CO_2_ drying, as demonstrated in **Figure** **7**.^77^ The resulting CNT–graphene hybrid aerogel showed high desalination capacity (633.3 mg g^−1^). In a much simpler process, a GO–CNT aqueous mixture was directly cryodesiccated, avoiding the formation of the hydrogel. This “sol–cryo” protocol developed by Sun et al. enabled the fabrication of ultralightweight hybrid aerogels with controlled densities, depending on the weight fraction of CNTs.^78^ The unique configuration of graphene sheet cell walls with CNT ribs as reinforcements guaranteed such an ultralight and stable all‐carbon structure. The ultralightweight aerogels were found to have outstanding elasticity, thermal stability, and adsorption capacity. When CNTs were entirely absent in the aerogels, the density could be extremely low. Interestingly, the achieved minimum density of the neat graphene aerogels (0.16 mg cm^−3^) was once seen as the lowest value for ultralight materials until the record was broken very recently by a lower value of 0.12 mg cm^−3^.^79^

### 3.2 Hybrid Structures with Polymers

Various graphene/polymer hybrid structures have been prepared via LbL assembly because of their functional groups for hydrogen bonding or electrostatic attraction. Such graphene/polymer hybrids with outstanding electrical and mechanical properties have indicated great promise in various applications such as SCs, conductive electrodes, and gas barriers. Though tremendous efforts have been made in using graphene as a nanofiller in polymer matrices, in this section, we focus on the self‐assemblies in which graphene and polymer are considered as individual building blocks.

For instance, the negatively charged GO and PEI with positively charged groups could form GO/PEI assemblies by virtue of apparent electrostatic interactions. Grunlan and co‐workers prepared GO/PEI on a polyethylene terephthalate (PET) film via the LbL assembly method for gas barrier application. It was noticed that a graphene/polymer assembly could provide a tightly packed architecture with insufficient gas diffusion channels.^80^ Chen et al. employed similar methods to construct gas barrier films using GO/PEI multilayered structures and demonstrated that both the pH for the GO suspension and the GO/PEI bilayer number had great influence on the oxygen barrier performance for the multilayered film,^81^ implying the intercalated structures of tightly oriented and stacked GO/PEI system. Enhanced mechanical properties were also reported by Grunlan and co‐workers for a PAA/PVP‐G system. PAA*_x_*/PVP‐G*_x_* layered films can also be obtained by sequential dipping a substrate, coated with a PEI primer layer, into PAA and PVP‐G aqueous solutions. This as‐prepared film showed increased elastic modulus by 322% (from 1.41 to 4.81 GPa), with a high light transmittance of ≈90%.^82^

**Figure 8:**
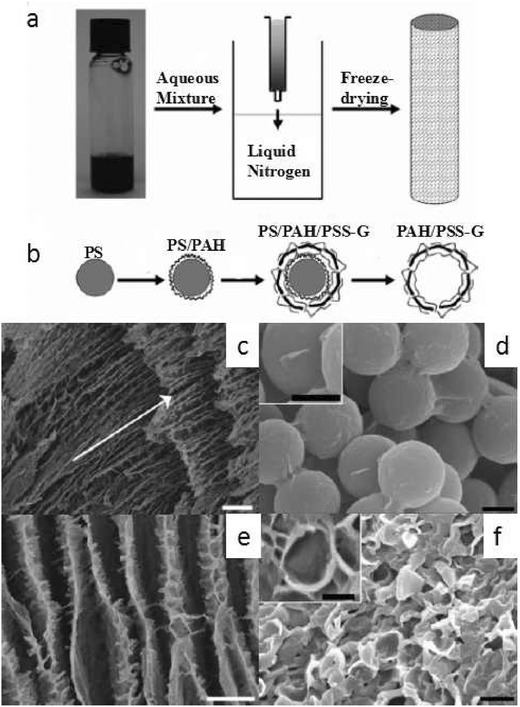
Schematics showing the fabrication of a) macroporous PSS‐G/PVA monoliths using ice‐segregation‐induced self‐assembly and b) hollow PSS‐G microspheres with the colloidal templating method. SEM images of c) longitudinal sections and e) cross sections of a PSS‐G/PVA freeze‐dried monolith. d) SEM images of PSS‐G‐coated PAH‐functionalized polystyrene beads (PS/PAH/PSS‐G), and the inset shows the curled edge of adsorbed PSS‐G sheet. f) SEM images of aggregated film of PAH/PSS‐G hollow microsphere. The inset in panel (f) shows a single hollow microsphere. Scale bar for panels (c) and (e) = 10 µm; panel (d) = 500 nm; and panel (f) = 1 µm. Reproduced with permission.^89^ Copyright 2009, Wiley.

Other polymers such as polyaniline (PANI),^83^, ^84^, ^85^ poly (dimethyldiallylammonium chloride) (PDDA),^86^ and PVA^87^ were also widely used in the LbL assembly of graphene/polymer hybrid films. For instance, Shi and co‐workers demonstrated an LbL assembly of electroactive graphene/PANI multilayered films with good transparency and conductivity yet without any supporting conductive transparent electrodes.^88^ By adjusting the deposition cycles of the multilayered films, one can readily modulate the film conductivity, the film thickness as well as the film transmittance. As for the 3D architecture of graphene/polymer composite, the most straightforward method is template‐directed assembly. Vickery et al. demonstrated the preparation of graphene/polymer hybrid structure with highly ordered 3D architectures by utilizing either ice or colloid particles as effective templates. A homogeneous aqueous solution of polystyrene sulfonate‐stabilized graphene (PSS‐G) sheets and PVA was injected into liquid nitrogen through an insulin syringe. The frozen sample can lock water inside the composite network. The removal of the ice template led to the formation of a PSS‐G/PVA aerogel.^89^ When using colloid particles as templates, PS beads were first modified with positively charged poly(allylamine hydrochloride) (PAH). After that, the modified PS was employed as the template to anchor the negatively charged PSS‐G onto the PS surfaces via electrostatic forces. The PS templates could be readily removed by toluene wash, ultimately generating hollow PSS‐G microspheres as shown in **Figure** **8**.^89^

Polymers can also act as the cross‐linker in the 3D graphene/polymer monolith to boost the mechanical properties of the composite system. Zhao et al. demonstrated the fabrication of a compressible and conductive graphene aerogels with introduction of conducting polymer polypyrrole (PPy), which functioned as an effective cross‐linker to yield the uniform dispersion of GO in aqueous solution for compensating the decrease in conductivity from conventional polymer cross‐linking.^90^ Such graphene aerogels prepared using the PPy as the cross‐linker were highly compressible with high conductivity. Even after deformation to 80% of the fracture strain, they could recover their original state without any apparent network collapse while retaining the electrical conductivity as high as ≈30 S cm^−1^.

### 3.3 Hybrid Structures with Metal or Metal Compounds

Self‐assembly of graphene or CMG with various metal or metal compounds has been widely studied.^91^, ^92^, ^93^, ^94^, ^95^ Inorganic nanoparticles can assemble on the CMG surfaces as particle nucleated/anchored on CMG substrates, with different characters and morphologies, resulting in hybrid structures with unique features inherited not only from the individual components but also emerged from the synergistic coupling of their components.^96^, ^97^, ^98^, ^99^, ^100^ The inherent properties of graphene or chemically modified graphene make it a suitable substrate for nanomaterials in many various applications.^101^, ^102^ In addition, graphene and nanoparticles could complement each other, and both sides of the 2D structured graphene were exposed to the solution, resulting in a large exposure of active surface area and a homogeneous distribution of nanoparticles. Among them, Pd,^103^ Pt,^104^ Au,^105^, ^106^, ^107^ TiO_2_,^108^, ^109^ quantum dots (QDs),^110^ Fe_3_O_4_,^111^ and so on have been successfully combined with chemically modified graphene, resulting a variety of different hybrid architectures.

Graphene‐based inorganic nanoparticle hybrids synthesized by LbL assembly are very common, which exhibit improved performance with respect to that of simply mixed hybrids. For instance, Zhou and co‐workers^112^ synthesized sandwich‐like hybrid nanosheets consisting of graphene‐wrapped SnO/SnO_2_ nanocrystals anchored on graphene by a facile LbL self‐assembly method. This unique double protection of graphene layers results in the hierarchical structure exhibiting good durability and rate performance when used in Li‐ion batteries (LIB).

Liu and co‐workers^110^ demonstrated well‐defined graphene–CdS quantum dots (GNs–CdS QDs) by sequential LbL self‐assembly. First, by in situ chemical reduction of GO sheets within the presence of cationic PAH, well‐dispersed polymer‐functionalized graphene nanosheets in aqueous solution were prepared. Subsequently, the resultant positively charged PAH‐modified GNs (GNs‐PAH) were assembled with negatively charged QDs to produce well‐defined composite films of GNs‐CdS QDs through a sequential LbL method. The alternating GNs−CdS QDs multilayered films, wherein the CdS QDs were uniformly spread over the 2D graphene nanosheets, showed great enhancement of photocatalytic and photo‐electrochemical activities upon visible light irradiation with respect to the pristine CdS QDs and graphene nanosheet films. It was suggested that all the enhancement lies in the judicious integration of CdS QDs with graphene nanosheets in an alternative fashion, thus maximizing the 2D structural merit of graphene nanosheets in GNs–CdS QDs hybrid films. The LbL‐assembly method was also used to fabricate other graphene‐based metal and metal compound nanostructures, such as Pd,^113^ Au,^114^ MoO_2_,^115^ and MnO_2_.^116^

**Figure 9:**
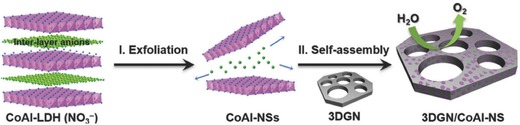
Schematic illustration of fabrication of 3D porous electrocatalyst, i.e., 3DGN/CoAl‐NS. Reproduced with permission.^117^ Copyright 2016, Wiley.

Graphene‐based composites can also be fabricated by solution‐based electrostatic self‐assembly, yielding a variety of readily dispersed and easily processable composites on a large scale by simply blending the aqueous dispersions of the assembling components, compared to the aforementioned LbL method. For example, Ping et al.^117^ produced a novel 3D graphene network‐based CoAl‐layered double hydroxides (LDHs) (3D GN/CoAl‐NS) nanocomposite through electrostatic self‐assembly of CoAl‐LDH nanosheets (CoAl‐NSs) with a single layer on a 3D GN. As shown in **Figure** **9**, the bulk CoAl‐LDH (CO_3_^2−^) was first prepared by a hydrothermal treatment. Subsequently, the bulk CoAl‐LDH crystal was immersed into a solution bearing an excess of anions to increase the interlayer distance, which accelerated the subsequent exfoliation of CoAl‐LDH.^118^ Finally, the single‐layer CoAl‐NSs were self‐assembled on the acid‐treated 3D graphene networks through the electrostatic adsorption. The achieved 3D GN/CoAl‐NS showed comparable or much superior performance and long‐term stability for oxygen evolution reaction (OER) in alkaline medium in comparison with most previously reported LDH‐based OER electrocatalysts. This method was also extended to produce CMG‐based hybrid structures in combination with other metal NPs,^119^, ^120^ metal oxide/hydroxide NPs,^121^, ^122^, ^123^ and QDs.^124^, ^125^

A GO or CMG aqueous solution can be assembled to form a 2D thin film by the orientation of the external force. Both vacuum filtration and flow‐induced self‐assembly approaches were based on this principle, and graphene/inorganic nanoparticle hybrids with ordered microstructures could also be prepared by the above methods, provided that the same solvent could be utilized for obtaining stable dispersion of assembled components. Recently, Li et al.^126^ proposed a new and effective approach to synthesize graphene/Fe_3_O_4_ composites with 3D structures by hydrothermal processing. First, the GO/Fe_3_O_4_ mixture suspension was deposited on an Ni foam by vacuum filtration, which was followed by freeze‐drying. The GO/Fe_3_O_4_ composites further underwent a plasma treatment to achieve simultaneous nitrogen doping and reduction. The as‐prepared 3D N‐doped graphene/Fe_3_O_4_ composite was used for a high‐performance SC electrode, showing a 153% improvement in specific capacitance compared to 3D graphene/Fe_3_O_4_ structures synthesized by the traditional hydrothermal method.

It is known that self‐assembly of nanoparticles on the graphene surface is a great challenge because of the lack of dangling bonds in the graphene plane. Yu and co‐workers^127^ reported the synthesis of graphene‐supported ultrafine MgH_2_ NPs with both homogeneous distribution and high loading percent by a hydrogenation‐induced solvothermal method. The proposed unique bottom‐up assembly strategy enabled strong coupling between graphene and MgH_2_ NPs as well as the homogeneous distribution of nanoscale particles, resulting in admirable H_2_ storage performance in graphene/MgH_2_ composites. On the other hand, the concept of assembling MgH_2_ NPs on graphene with high loading also represents a viable path for fabricating various nanostructured nanocomposites with potential applications in energy‐related fields. This strategy was universal and was also employed to fabricate other graphene‐based metal or metal compound composites, such as TiO_2_,^121^, ^128^ Au nanoparticles NPs,^129^ CdS QDs,^130^ and GaN layers.^131^

Another successful fabrication method for graphene/inorganic particle hybrids is built on in situ growth and assembly of metal nanoparticles on functionalized graphene. However, metal nanoparticles are usually located randomly on the graphene surface. Takeuchi and co‐workers^95^ demonstrated that gold (I) cyanide (AuCN) could be grown on pristine GN as nanowires via a self‐organized process in a water solution at ambient condition. The AuCN nanowires could align themselves along the zigzag lattice direction of graphene, which originated from the interaction with the gold atom and the lattice match suggested by the first‐principles calculations. Dangling bonds of damaged graphene,^132^, ^133^ vapor‐phase deposition at high temperature,^134^, ^135^, ^136^, ^137^, ^138^, ^139^, ^140^, ^141^, ^142^, ^143^, ^144^, ^145^, ^146^, ^147^, ^148^ and intermediate seed materials^132^, ^148^, ^149^, ^150^ have been used to produce the hybrid of pristine graphene/inorganic nanostructure; however, the formed inorganic nanostructures were randomly oriented or poorly aligned on pristine graphene. This work paves a new way for precisely assembling inorganic nanomaterials on pristine graphene.^93^, ^135^, ^136^, ^137^, ^138^, ^139^

Zhang and co‐workers^141^ recently demonstrated that the well‐dispersed mesoporous TiO_2_ nanocrystals with (001) facets could be assembled onto a graphene aerogel surface (TiO_2_/GAs) by a one‐pot hydrothermal treatment. In this process, the glucose functioned as not only the linker but also the face‐regulating agent to yield (001) facets as well as a mesoporous TiO_2_ structure. The resulting TiO_2_/GAs composite showed high specific capacity and high recyclability of photocatalytic activity for methyl orange pollutant when used for photocatalysis and Li‐ion battery. Other metal or metal oxides or hydroxides (e.g., TiO_2_, SnO_2_, NiOH, and MnO_2_) have also been assembled on graphene, forming hybrids with well‐controlled nanostructures by using a similar strategy.^142^, ^143^, ^144^, ^145^, ^146^, ^147^

These graphene‐based hybrid nanostructures exhibit promising performance in various applications because of the strong interaction and synergistic effects between graphene and the metal/metal compounds. Moreover, graphene as an ideal support material can greatly enhance the durability and stability of the hybrid materials. To broaden practical applications of self‐assembled graphene/metal or metal hybrid structures, their size and morphology need to be further optimized.

## 4 Applications of Self‐Assembled Graphene‐Based Architectures

Self‐assembly of graphene‐based structures with unique morphologies and tunable compositions has paved the way for a wide variety of potential applications such as electronic, optoelectronics, energy storage and conversion, electrocatalysis, photocatalysis, and environment. Graphene‐based architectures with the versatility of their self‐assembly behavior are capable of obtaining desirable hybrids and macroscopic architectures aiming for particular functions and properties. Certainly, CMGs within these architectures or superstructures do not only function as a 2D scaffold or support featuring atomic thickness, high conductivity, and a large specific surface area but also frequently provide a synergistic effect to the active components affording further performance improvement in various devices. In this section, we highlight some typical examples of the self‐assembled graphene‐based architectures applied in electronics, optoelectronics, energy storage and conversion, catalysis, and environment to indicate the diverse directions of this research area.

### 4.1 Electronics and Optoelectronics

The highly conductive, transparent, and flexible graphene thin films are anticipated to be utilized in developing cheap and flexible transparent graphene‐based electrodes to compete with conventional indium tin oxide electrodes. Various self‐assembly methods such as flow‐induced self‐assembly, LbL, and LB techniques are effective in constructing transparent conductive electrode with low cost and tunable properties compared with conventional metal/metal oxide electrode. For instance, by taking advantage of graphene over conventional metals for the anode, Wu et al. fabricated devices based on a graphene/P3HT/Si nanowire array, achieving a power conversion efficiency (PCE) of 9.94%, which is much higher than that of Cu and Au film‐based devices with 5.3% and 7.8% power conversion efficiency. The enhanced device performance is attributable to a much flatter transmittance curve of the graphene films in the whole spectrum relative to metallic films. Moreover, the high transmittance, along with high conductivity and adjustable work function, makes graphene an appealing choice for uses in transparent conductors,^148^, ^149^ photosensitizers,^150^ and channels for charge transport^151^ in solar cell applications. In addition, self‐assembled CMG structures could function as electron acceptors or hole‐extraction layers (HEL) for use in polymer solar cells (PSCs). For example, the bulk heterojunction solar cells using the hybrid fiber assembled from dioctyl‐perylenedicarboximide (dioctyl‐PDI) and rGO as an electron acceptor and poly(3‐hexylthiophene) (P3HT) as an electron donor exhibited a PCE of 1.04%, superior to those using pure dioctyl‐PDI and the dioctyl‐PDI/rGO mixture. The improved performance arises from the efficient exciton dissociation at the donor/acceptor interface together with fast charge transport within the nanofibers.^152^ Gao et al. demonstrated the use of GO thin films as the HEL in inverted PSCs. The homogeneous GO layer was assembled onto the P3HT:[6,6]‐phenyl‐C61‐butyric acid methyl ester active layer. The resulting inverted PSC with desirable GO layer thicknesses of 2–3 nm yielded a PCE of 3.60%. This value is on a par with that of traditional poly(3,4‐ethylenedioxythiophene) doped with poly(styrene sulfonate)‐based inverted device. It was suggested that GO bearing oxygen‐carrying groups including enolic and carboxylic groups with a relatively high proton content could dope P3HT at the active layer surface, which favored the formation of an Ohmic contact between the top metal electrode and the active layer, and thus results in significantly improved device performance.^153^

**Figure 10:**
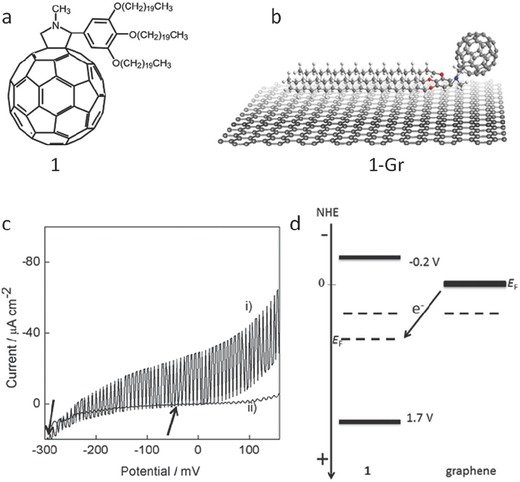
a) Chemical structure of alkylated‐C60 (denoted as 1). b) Schematic illustration of formation of 1‐graphene (1‐Gr) and the proper affinity of 1 toward the surface of graphene. c) Photovoltammograms of 1‐Gr/FTO (i) and 1/FTO (ii) electrode systems under chopped white light. d) Proposed mechanisms for *E*_F_ shift of 1 in the presence of graphene. Reproduced with permission.^154^ Copyright 2015, Wiley.

Nakanishi and co‐workers demonstrated an optoelectronically active assembly of alkylated‐C60 and graphene through direct exfoliation of graphite in organic solvents within the presence of 3,4,5‐tris(eicosyloxy)phenyl substituted *N*‐methyl[60]fullero‐pyrrolidine through noncovalent interactions (**Figure** **10**).^154^ The resulting graphene/alkylated‐C60 assembly yielded around 270‐fold higher photocurrent than that of the parent C60 derivative (Figure 10). This result implies that the graphene/alkylated‐C60 assembly can function as excellent electron accepting/charge transport materials for highly efficient photovoltaic devices.^154^

LbL assembly in conjunction with other wet‐chemical methods such as dielectrophoresis can be utilized to produce GO/rGO–nanoparticle hybrids with multilayered structures. For instance, Li and co‐workers reported layered thin films assembled from CdS quantum dots and rGO sheets which involved the dielectrophoretic deposition of an rGO thin film on a targeted substrate with subsequent in situ growth of a CdS layer through a wet‐chemical method. The photovoltaic devices constructed using these hybrid films showed an open‐circuit voltage of 0.68 V when the rGO/CdS bilayer number reached 2 or more. At eight bilayers, the device yielded a short‐circuit photocurrent density of 1.08 mA cm^−2^ and a high incident photocurrent efficiency of 16%. Such enhanced device performance should arise form unique layered structures of the hybrid films.^155^ By LbL assembly, Yao et al. prepared TiO/GO multilayered hybrid films. After reduction of GO, the resulting hybrid films exhibited improved photoresponse as well as excellent reversibility and stability under ambient conditions.^156^

Self‐assembled graphene hybrid structure can also be used in high‐performance FETs, which require a thin film with smaller toughness and higher reduction degree. Hybrid films of (PAH/GO/PAH/PW)*_n_* with a different number of layers (*n*) of GO were fabricated via the electrostatic LbL assembly, and subsequent photoreduction under UV light converted GO to rGO in the composite film.^157^ FET devices constructed using graphene‐based hybrid films presented typical ambipolar characteristics with good hole and electron transport properties. The hole‐dominated transport feature was credited to the electron‐trapping effect of the cage‐type structure of H_3_PW_12_O_40_ (PW). The on/off ratios for these FET devices were calculated to be about 1.1–2.0 for the films with different GO layers. By self‐assembly of graphene single crystals induced by a mutual electrostatic force between the neighboring crystals with the assistance of the airflow‐induced hydrodynamic forces, Fu and co‐workers produced a new graphene superordered structure (GSOS) with adjustable periodicity in the spacing and remarkable uniformity in size and orientation. Such a simple yet efficient strategy inherits the merit of the conventional self‐assembly approach. A back‐gated FET array constructed using GSOS exhibited the device mobility of 3882 ± 896 cm^2^ V^−1^ s^−1^, which signifies high quality of the resulted GSOS.^158^ Halik and co‐workers assembled oxofunctionalized graphene/polymer hybrid films for memory devices, which can be operated at a low voltage of 3 V. It was found that the coating layer of ≈5 nm is mandatory to afford the relative low operation voltage of the device.^159^ More recently, self‐assembled 3D free‐standing, hollow, polyhedral graphene was reported, demonstrating interesting optoelectronic properties. It was found that such a unique 3D graphene polyhedron induced uniform plasmon–plasmon couplings at each of the faces, while abundant 3D plasmonic hybridization behavior triggered a high degree of volumetric light confinement.^160^

### 4.2 Photocatalysis

**Figure 11:**
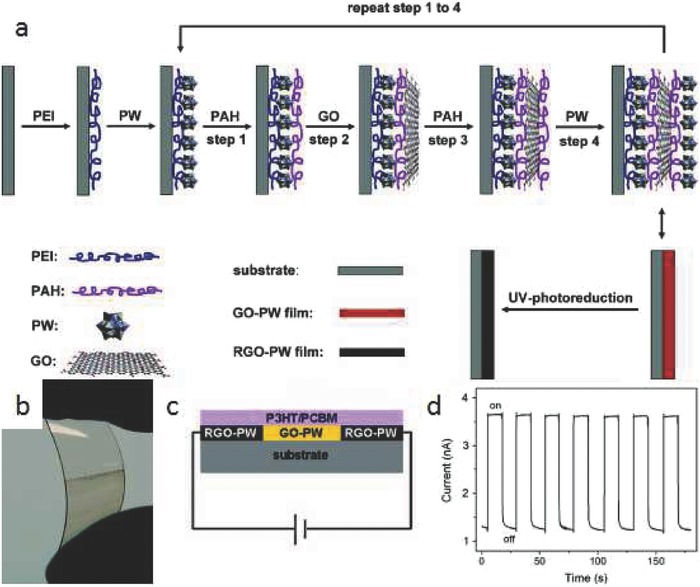
a) Schematic illustration of the fabrication procedure of rGO–PW multilayered films. b) Optical images of a (PAH/GO/PAH/PW)_6_ multilayered film prepared on flexible PET substrate after 6 h of UV photoreduction. c) Cross section of the photodetector device. d) Photocurrent response of the photodetector versus time under chopped irradiation, at a bias voltage of 10 V. Reproduced with permission.^157^ Copyright 2014, American Chemical Society.

**Figure 12:**
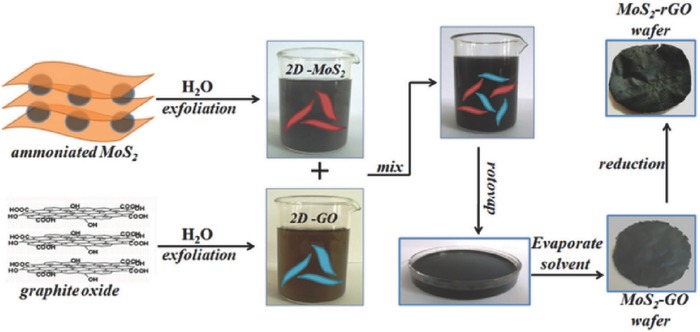
Schematic representation of the synthesis of MoS_2_–rGO hybrid paper. Reproduced with permission.[[qv: 161e]] Copyright 2017, Elsvier Ltd.

Compared with the conventional active materials, the CMG‐based hybrid structures have been proved to be efficient catalyst in many photocatalysis systems where CMG sheets with anisotropic 2D structure, large specific surface area, and superb conductivity, are regarded as ideal host for anchoring catalytic materials.[[qv: 161a–e]] For instance, due to its strong oxidizing power, long‐term thermodynamic stability, and low toxicity, TiO_2_ was recognized as one of the most promising photocatalytic materials. As expected, the combination of TiO_2_ and graphene sheets could also yield hybrid nanostructures with high photocatalytic activity. As a typical example, 2D hybrid structures of GO sheets and rod‐like TiO_2_ particles(TiO_2_/GO) were produced via the interfacial self‐assembly. The photodegradation of methylene blue (MB) for TiO_2_/GO was evaluated by comparing with that of the blend of GO sheets and commercial TiO_2_ particles.^161^ By monitoring the UV–vis absorption spectrum of MB in aqueous solution, the complete photodegradation of MB (10 ppm) for TiO_2_/GO was achieved with 15 min of UV‐light irradiation. In contrast, only 70% of MB was found to be decomposed in the case of oleic acid stabilized TiO_2_ nanorods. Such good photocatalytic performance for TiO_2_/GO can be credited to the use other GO scaffold, which is able to inhibit charge recombination during the photocatalytic process. For the photoreduction of nitroaromatic compounds, Xiao et al. constructed an alternating GN–CdS QDs multilayered film through the LbL self‐assembly of water‐soluble cationic PAH‐modified graphene sheets and ionic CdS QDs (**Figure** **11**). The photocatalytic reduction of organic pollutant over GN–CdS QD composite films showed over 90% generation rate for 3 h under visible‐light irradiation (λ > 420 nm). Such performance was superior to that of CdS QD films (≈60% for 3 h). The good photocatalytic performance for GNs‐CdS QD hybrid films comes from the presence of the graphene sheets that not only act as an efficient electron collector but also facilitate the transport for the photoexcited electron from CdS QDs, thus rendering an effective suppression of the photoexcited electron–hole recombination.^110^ Apart from TiO_2_ and CdS QDs, hybrid structures formed by graphene and layered transition metal dichalcogenides have also been given attention due to the fact that this combination showed interesting physical properties suitable for potential applications in photocatalysis.[[qv: 161c–e]] For example, Rajamathi and co‐workers reported a large free‐standing MoS_2_–rGO hybrid papers via a simple exfoliation–costacking method involving evaporation under ambient conditions (**Figure** **12**). Such hybrids exhibit good performance as photocatalysts for degradation of organic dyes (complete degradation of 10 mg L^−1^ MB in 30 min).[[qv: 161e]]

### 4.3 Electrochemical Energy Systems

Electrochemical energy storage conversion systems are of great significance in fulfilling the increasing demand for high‐performance energy source throughout the world, with great potential in electric vehicles, portable electronics, and so on. The excellent conductivity and high surface area of the graphene hybrid structure make it an ideal substitute of conventional metal electrode for energy devices such as SCs, Li‐ and Na‐ion batteries, Li–S and Li–O_2_ batteries.

The highly porous structure and electrical conductivity of the graphene hybrid superstructure enable better ion diffusion and electron transport in bulk electrodes, which are desirable for macroscale and microscale SC applications. In our previous work, we reported the sequential self‐assembly of large‐area hybrid films of functionalized CNT, and graphene and CNTs via electrostatic attraction. A rectangular‐like cyclic voltammogram was achieved at a high scan rate of 1000 mV s^−1^ while yielding an specific capacitance up to 120 F g^−1^, which signifies rapid charge/discharge behavior in formed hybrid film electrodes. Such good capacitive behavior of the hybrid film electrode was attributed to the conductive interconnected porous network carbon structures for rapid ion diffusion.^86^ High electrical conductivity is another essential requirement for SC electrodes. Pham et al. synthesized the CNT/graphene films via self‐assembly based on Coulombic interaction. The resulting flexible graphene/CNT films exhibited high electric conductivity up to 394 S cm^−1^. SCs fabricated with these films showed a specific energy density of 110.6 Wh kg^−1^ at 400 kW kg^−1^.^161^ The CMGs can also be utilized as a conductive scaffold to anchor various electrochemical active components such as conductive polymer, transition metal oxides and hydroxides, forming new hybrid structures for high‐performance pseudocapacitors.^162^, ^163^, ^164^, ^165^ As a representative example, Ni(OH)_2_ with an ultrahigh theoretical capacitance of 2600 F g^−1^ was assembled onto rGO sheets, which delivered an ultrahigh specific capacitance up to 1335 F g^−1^ at 2.8 A g^−1^ and a long cycling life. Such excellent performance was ascribed to the synergistic effect of conductive graphene and highly crystalline Ni(OH)_2_ nanoplates.^166^

More recently, Gogotsi and co‐workers produced a flexible and conductive MXene/rGO SC electrode. Through electrostatic assembly between negatively charged titanium carbide MXene sheets and positively charged rGO functionalized by PDDA, it was found that rGO sheets were inserted in between MXene layers, prohibiting the restacking of MXene sheets and resulting in significantly increased interlayer spacing. This will accelerate electrolyte ion diffusion and enable more accessible active sites. Thus, the produced self‐supporting MXene/rGO‐5 wt% SC electrode yielded a volumetric capacitance as high as 1040 F cm^−3^ at 2 mV s^−1^, a high rate capability and a long cycle life. Furthermore, the constructed binder‐free SC gave an ultrahigh volumetric energy density up to 32.6 Wh L^−1^.^167^

Self‐assembled 3D CMG‐based architectures were promising electrode materials for flexible or deformation‐tolerant SCs. Duan and co‐workers produced a 3D graphene‐based hydrogel by a hydrothermal process. The resulted hydrogel can be used for advanced flexible all‐solid‐state SCs. The optimized SCs exhibited remarkable capacitive performance, including a gravimetric specific capacitance of 186 F g^−1^, an areal specific capacitance of 372 mF cm^−2^, very small leakage current (10.6 µA), superb stability, and excellent mechanical flexibility. The extraordinary performance was attributed to a highly interconnected porous network structure with high electrical conductivity and mechanical robustness.^168^ Qu and co‐workers explored a strategy to construct a CMG‐based hydrogel that integrated 3D graphene with polypyrrole by premixing pyrrole monomer with GO sheets for hydrothermal processing, followed by the electropolymerization. The produced composite foams can be utilized for compression‐tolerant capacitors, which yielded large specific capacitances without significant change when undergoing long‐term compressively loading/unloading cycles.^169^

Due to continued miniaturization of wearable/portable electronics, micro‐supercapacitors (micro‐SCs) in the forms of either fibers or thin films are particularly attractive as miniature energy storage units. Recently, we fabricated a new kind of 1D macroscopic rGO/CNT composite fiber by space‐confined assembly. Such self‐assembled hybrid fibers can be used to build fiber‐based micro‐SCs, exhibiting an energy density as high as ≈6.3 mWh cm^−3^, on par with 4 V/500 µAh thin‐film lithium batteries, together with a power density much higher than that of batteries as well as an ultralong cycle life and good flexibility. The remarkable performance was attributed to unique self‐assembled fiber structure combining high conductivity, large surface area, and desirable porosity. Following on our work, a series of various self‐assembled CMG‐based fibers have been employed to construct fiber SCs, demonstrating excellent electrochemical performance and great potential of CMG‐based fiber electrodes for advanced microscale power sources. In addition to fiber micro‐SCs, an in‐plane micro‐SC was fabricated using stacked‐layered heterostructure films from thiophene nanosheets and exfoliated graphene reported by Feng and co‐workers The optimized micro‐SC can work at an ultrahigh rate of up to 1000 V s^−1^ with a landmark areal capacitance of 1.30 mF cm^−2^ and volumetric capacitance up to 123 F cm^−3^ at 100 V s^−1^ as well as excellent flexibility under various bending states.^163^

Apart from their use in SCs, self‐assembled CMG‐based hybrid structures are found to be promising electrode materials for lithium‐ion battery (LIB), since it possesses controllable thickness, large aspect ratio, and high surface area, which favor the electrolyte access and fast ion/electron diffusion. Self‐supporting and flexible SnO_2_–rGO hybrid films were prepared by in situ self‐assembly which could be utilized as efficient electrodes LIB.^145^ The composites with 40 wt% rGO showed a specific capacity of up to 760 mA h g^−1^ at 0.008 A g^−1^, which approaches the theoretical value of 780 mA h g^−1^. SnO_2_ is regarded as a promising high‐capacity anode material for LIB, but further commercialization is hindered by the poor cycling stability.^170^ By contrast, the reported SnO_2_–rGO composites exhibited good stability at various current densities with no apparent drop in specific capacities. This suggests excellent space‐confinement effect and electrical contact between rGO sheets and SnO_2_ in formed layered hybrid structures. Recently, Zhao et al. explored an in situ reduction method for synthesizing SnO_2_ quantum dots@GO by the oxidation of Sn^2+^ and the GO reduction. Such composites showed a capacity retention of 86% obtained even after 2000 cycles at 2 A g^−1^.^170^ Effective combination of ultrafine SnO_2_ quantum dots on conductive graphene sheets provides abundant active sites for high lithium storage capacity, and the confinement of SnO_2_ quantum dots in graphene sheets inhibits the aggregation of each particle for high‐rate cycling stability, which also verified the great potential of the graphene assemblies for the fabrication of advanced LIBs. Other active materials such as Mn_3_O_4_,^171^ SnO_2_/Fe_2_O_3_,^172^, ^173^ CoO^174^ and V_2_O_5_ 175 could also be incorporated into graphene scaffold via simple self‐assembly methods.

3D networks of graphene hybrids were also demonstrated as anode materials for LIBs. For example, Bai et al. demonstrated the synthesis of a porous 3D graphene hydrogel with an embedded Si/SiO*_x_* core–shell structure (Si@SiO*_x_*/GH) through a solution‐based self‐assembly process. Such 3D hybrids afforded a stable capacity up to 1020 mA h g^−1^ at 4 A g^−1^ and 1640 mA h g^−1^ over 140 cycles at 0.1 A g^−1^. The superb electrode performance for Si@SiO*_x_*/GH can be attributable to the following aspects: (1) the conductive graphene matrix is capable of accommodating the volume change of Si nanoparticles; (2) the porous 3D architecture with large specific surface area allows fast Li‐ion diffusion as well as easy electrolyte penetration.^176^

**Figure 13:**
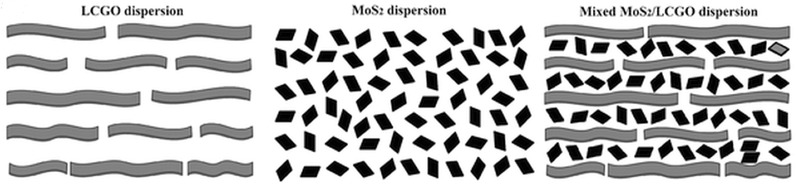
Schematic representation of those three dispersions. Reproduced with permission.^177^ Copyright 2017, Wiley.

More recently, Wallace and co‐workers fabricated a flexible, self‐supporting 3D porous MoS_2_–rGO film with a 3D porous structure via a one‐pot self‐assembly, gelation, and subsequent reduction (**Figure** **13**). The self‐assembled MoS_2_–rGO film containing 75 wt% of MoS_2_ showed a high capacity of up to 800 mA h g^−1^ at 100 mA g^−1^ with excellent rate performance, and superb cycle stability with no obvious capacity fading over 500 cycles at 0.4 A g^−1^.^177^

Through a facile hydrothermal coassembly route, Chen and co‐workers prepared a 3D porous composite foam made up of graphene/Sb_2_S_5_ nanoparticles. Such assembled 3D graphene‐based structures can serve as efficient anode materials for Na‐ion batteries without using a binder or current collector, which yielded a high capacity of 845 mA h g^−1^ at 0.1 A g^−1^, a long cycling life with a 91.6% capacity retention over 300 cycles at 0.2 A g^−1^, and superb rate performance (525 mA h g^−1^ at 10 A g^−1^). The excellent electrochemical performance is credited to rapid Na^+^ ion diffusion from the ultrasmall‐sized nanoparticles and excellent charge transport between the active component and 3D porous graphene foam structure.^178^ By combining a templated method and a self‐assembly process, a 3D porous structure consisting of rGO nanowire on 3D graphene foam was used as the anode material for Na‐ion batteries, demonstrating a reversible capacity of more than 301 mA h g^−1^ without capacity drop over 1000 cycles.^179^

Manthiram and co‐workers produced 3D porous nitrogen‐ and sulfur‐codoped graphene sponges by a hydrothermal assembly and utilized them as self‐standing electrode for Li–S batteries, which exhibited a large specific capacity of 1200 mA h g^−1^ and a high‐rate capacity of 430 mA h g^−1^ at 0.2 C rate accordingly with superior cycling stability. The excellent electrode performance lies in the combination effect of the physical adsorption of lithium polysulfides onto 3D self‐assembled porous graphene network and the chemical adsorption of polysulfides over N and S sites of graphene.^180^ Xu and co‐workers demonstrated the use of self‐assembled graphene/poly (anthraquinonyl sulfide) composite aerogel as a self‐supporting flexible cathode for rechargeable Li‐ and Na‐ion batteries. The obtained 3D aerogels yielded a capacity of 156 mA h g^−1^ at 0.1 C with good rate performance (102 mA h g^−1^ at 20 C) for LIBs, and demonstrated a high‐rate capability (72 mA h g^−1^ at 5 C) and an ultralong stability (71.4% capacity retention over 1000 cycles at 0.5 C). The excellent cathode performance for both Li‐ and Na‐ion batteries was ascribed to the rapid redox kinetics and electron transport within 3D porous graphene network.^181^

3D graphene aerogels decorated with Ru particles (Ru‐GAs) showed the pore volumes of 2.8 and 14.1 cm^3^  g^−1^ below and above a critical pore size of 100 nm, which could be used as a free‐standing cathode in an Li–O_2_ battery. The produced cathode yielded a high capacity of >12 000  mA h g^−1^ and superb cycling stability, which was ascribed to the 3D porous structure, rich active sites with Ru particles, and excellent chemical stability.^182^

### 4.4 Electrocatalysis

The electrochemical oxygen reduction reaction (ORR) is the pivotal step for various next‐generation energy conversion systems including fuel cells, metal–air batteries, and certain electrolyzers.^183^, ^184^ Even though Pt‐based catalysts have been commercially available, their high cost and poor durability still hampered the large‐scale application of such catalysts. Non‐noble metal/metal oxide‐decorated graphenes have received much attention in pursuit of more cheap and efficient electrocatalysts bearing comparable or even superior catalytic performance relative to the conventional Pt‐based catalyst. For instance, Co/CoO@graphene hybrid exhibited superior performance for the ORR in a 0.1 m KOH by anchoring monodispersive Co/CoO nanoparticles onto a graphene surface by adopting a solution‐based self‐assembly strategy.^185^ Previous investigation regarding the interaction between graphene and metal has shown that the difference in the Fermi levels of graphene and the metal tends to lead to the charge transfer occurred at the graphene/metal interface.^186^ Improved catalytic performance of the NPs/graphene hybrid could be ascribed to the charge transfer, as demonstrated in various graphene‐supported catalysts such as Co_3_O_4_/graphene,^187^ Fe_3_O_4_/graphene,^188^ Co*_x_*S_1−_ *_x_*/graphene,^189^ and MnCo_2_O_4_/graphene^190^ for ORR in alkaline or acid media.

The OER and hydrogen evolution reaction (HER) are also crucial electrochemical processes for various energy conversion systems. Different from substrate‐assisted electrodes, self‐assembled graphene hybrid nanostructures offer new opportunities for fabricating self‐standing electrodes with tunable microstructures and mechanical performance for mass transport and catalytic performance enhancement. Qiao and co‐workers presented the first N,O‐dual doped graphene‐CNT (NG‐CNT) hydrogel film by LbL assembly of GO and CNTs followed by N‐doping. The resultant free‐standing hydrogel film exhibited prominent OER performance, superior to the benchmark noble metal oxide (IrO_2_) and some reported transition‐metal catalysts.^191^ Furthermore, another simple vacuum filtration method has been demonstrated to integrate 2D porous g‐C_3_N_4_ nanolayers and N‐doped graphene into a free‐standing film featuring porous g‐C_3_N_4_ with highly exposed active sites and a desirable porous structure. The porous structure in the electrode not only provides large accessibility to the active centers but also facilitates the mass transport during the reactions. With the above benefits, this self‐standing electrode acted as a remarkably active HER catalyst, showing a low overpotential (80 mV) at 10 mA cm^−2^ with a small Tafel slope (49.1 mV dec^−1^) in 0.5 m H_2_SO_4_.^192^ Cheng and co‐workers. reported the fabrication of CoS_2_/rGO‐CNT hybrid nanostructures via an efficient hydrothermal process combined with vacuum filtration self‐assembly. The as‐produced conductive and robust film was evaluated as HER electrocatalysts. The unique CoS_2_/rGO‐CNT hybrid architectures led to a good HER activity of 142 mV overpotentials at 10 mA cm^−2^ and high durability due to synergistic effect between individual components.^193^ Other graphene‐based hybrid nanostructures such as MoS_2_ nanoflower/rGO paper and MoS*_x_*/N‐G can also be easily achieved via self‐assembly,^194^, ^195^ showing great potential for high‐performance electrocatalysts.

### 4.5 Water Treatment

Water pollution is harmful to the environment and human's health. Nanofiltration, possessing low energy cost and high efficiency, is regarded as a promising technology for the water purification. Due to diverse advantages over conventional materials such as availability, environmentally friendliness, and low cost, CMG‐based structures will provide superior applications in different water treatment such as dyes adsorption/degradation from water.

Single‐layered GO (SLGO) is regarded as a new generation of membrane material for high efficient water purification. To develop high‐performance GO‐based nanofiltration membrane, it is critical to achieve the trade‐off between selectivity and flux. Yu and co‐workers demonstrated that self‐assembly of SLGO by simply controlling deposition rate can break the selectivity/flux trade‐off. It was found that self‐assembled GO membranes, produced at slow deposition rate, showed dramatically enhanced salt rejection, while counterintuitively possessing 2.5–4 folds higher water flux than those of membranes derived at fast deposition rate. This finding is of significance for designing/regulating interlayered structure of ultrathin GO membranes by simply tuning SLGO deposition rate for highly efficient water treatment.^196^

By vacuum filtration, Chen and co‐workers created MWCNT/rGO hybrid membranes, which exhibited enhanced water permeability and membrane selectivity. When intercalating MWCNTs with the diameter of 10 nm within rGO membranes, the permeability of water was found to reach 52.7 L m^−2^ h^−1^ bar^−1^. This value is about 4.8 times higher than that obtained for original rGO membrane and 5–10‐folds higher than those for many commercial membranes. Furthermore, the membrane achieved almost 100% rejection rate for three various types of organic dyes. It was suggested that the nanotube inner wall could act as spacers, and nanosized rGO sheets exfoliates on the outer walls, which is interconnected with the assimilated rGO sheets to impart superb stability to the membrane.^197^

Tang and co‐workers produced a ZIF‐8 metal‐organic framework/rGO hydrogel via self‐assembly, in conjunction with the combination effects of cross‐linking by metal ions and chemical reduction. Upon freeze‐drying, the resulting hydrogel was converted into the ZIF‐8/rGO aerogel, which exhibited large absorption capacity and high cycling stability for organic solvents and oil. This was attributed to its super hydrophobic properties and large specific surface areas. Besides, the corresponding hydrogel afforded photocatalytic dye degradation capability, together with superb water purification performance for effective removal of heavy metal ions, toxic dyes, and benzo pollutants.^198^

3D graphene and graphene/CNT sponges were also demonstrated as absorbers for a variety of water pollutants due to their large specific surface area and good chemical stability in organic solvents.^78^, ^199^ Bi et al. reported an autoclaved‐reduction‐induced self‐assembly of rGO sponge with absorption capacity for 20–85 times of its own weight.^199^ Sun et al. fabricated an rGO–CNT sponge by freeze‐drying the GO/CNT solution with subsequent hydrazine vapor reduction. Such sponges showed great elasticity, high hydrophobicity, and ultralow density (a contact angle of 133° and a density of 0.16 mg cm^−3^). These features allowed the sponge to reach a significant absorption capacity of 215–743 g g^−1^ for various pollutants.^78^

## 5 Conclusions and Outlook

Thanks to their unique 2D structures, large specific surface area, and excellent physical and chemical properties, CMGs have been considered as the most appealing building blocks for “bottom‐up” self‐assembly, facilitating hierarchical and anisotropic organization of CMGs at different length scales with other inorganic or organic functional species by virtue of noncovalent forces. Meanwhile, self‐assembly of CMGs has been identified as one of the most attractive bottom‐up strategies for exploiting a variety of unprecedented carbon materials with tunable microstructure, designed functions and properties that are compatible with conventional processing methods. Self‐assembly derived processing methods and CMG materials are often featured by a variety of advantages. First, the assembled architectures have diverse interface‐directed morphologies ranging from 1D fibers, 2D films, to 3D porous network, which are suitable for many applications, such as flexible/compressible membrane‐like device. Second, the microstructure and surface/interface chemistry of the formed CMG‐based materials can be easily tuned, favorable for the controlled functionalization of materials for the targeted applications. Third, the self‐assembly of CMG renders numerous opportunities for hybridization with a wide variety of inorganic and organic components to construct homogeneous hybrid architectures for various applications. More importantly, self‐assembled CMG‐based materials with well‐defined structures have shown great potential in producing advanced functional systems for various technological fields such as electronics, optoelectronics, energy storage and conversion, catalysis, and water treatment.

Despite great advances in this research area of the self‐assembly of CMGs, at least the following several issues require further studies. First, it still remains a huge challenge to prepare CMGs with a defined shape and fine control of their surface chemistry, sizes, compositions, and monodispersity, since the structural variability and complexity of CMGs pose major impediments to afford the uniformity of self‐assembled structures for their large‐scale fabrication and real‐world applications. Meanwhile, it is a demand to develop new exfoliating strategies to achieve novel CMG building blocks with the well‐defined shape and desirable functional groups. Second, self‐assembly mechanism of CMGs and the structure–property correlation of self‐assembled CMG architectures lacks full understanding, yet this aspect is critical for effective control over the self‐assembly of CMGs as well as the design and preparation of functionalized CMG materials. In‐depth studies on the basic principles and theories of self‐assembly are urgently required to render guidance for further improve the performance and broaden the applications for CMG‐based structures. Third, it is necessary to explore more functional building blocks and integrate them into CMG‐based hybrids with unique structures, properties, and functions via self‐assembly. Particularly, the homogeneous hybridization is paramount for the preparation of CMG‐based functional materials. Finally, cost‐effective and upscalable assembly protocols are needed for the practical applications of the assembled materials. Combining multiple disciplines such as chemistry, physics, and materials science, it is optimistically anticipated that more and more self‐assembled CMG‐based architectures, superstructures, and functional devices with unprecedented properties, functions, and applications will be realized in the near future for real‐life money‐making applications. By taking into account the developments that have been made involving graphene, it can be said that this 2D “wonder material” and its self‐assembled functional architectures are heading toward a wonderful future.

## Conflict of Interest

The authors declare no conflict of interest.
